# Maternal and child health service readiness among primary health care facilities in Ekiti, Nigeria

**DOI:** 10.4102/phcfm.v14i1.3535

**Published:** 2022-08-19

**Authors:** Esther O. Oluwole, Adeyinka Adeniran, Chisom F. Chieme, Omobola Y. Ojo, Modupe R. Akinyinka, Marcus M. Ilesanmi, Babatunde A. Olujobi, Omowunmi Q. Bakare

**Affiliations:** 1 Department of Community Health and Primary Care, Faculty of Clinical Sciences, College of Medicine, University of Lagos, Surulere, Nigeria; 2 Department of Community Health and Primary Health Care, Faculty of Clinical Sciences, Lagos State University College of Medicine, Ikeja, Nigeria; 3 Department of Community Health and Primary Health Care, Faculty of Clinical Sciences, Lagos State University Teaching Hospital, Ikeja, Nigeria; 4 Petra Global Consulting, Ilasamaja, Nigeria; 5 Department of Community Medicine and Primary Care, Faculty of Public Health, Federal Medical Centre, Abeokuta, Nigeria; 6 Department of Planning, Research and Statistics, Primary Healthcare Development Agency, Ekiti State Primary Healthcare Development Agency, Ado Ekiti, Nigeria; 7 Department of Board and Oversight Management, Primary Healthcare Development Agency, Ekiti State Primary Healthcare Development Agency, Ado Ekiti, Nigeria

**Keywords:** maternal and child health, primary health care facilities, service readiness, Ekiti State, Nigeria

## Abstract

**Background:**

The availability of adequate infrastructure, diagnostic medical equipment, medicines and commodities and well-trained medical personnel are essential for the effective delivery of health care services.

**Aim:**

This study assessed maternal and child health (MCH) services’ specific readiness by type and location of the health facility and compared the readiness between urban and rural primary health care (PHC) facilities in Ekiti State, Nigeria.

**Setting:**

The study was conducted amongst the heads (officers in charge) of PHC facilities in Ekiti State, Nigeria between August 2020 and October 2020.

**Methods:**

A descriptive cross-sectional study in which all PHC facilities were conducted and data were collected with the aid of the Service Availability and Readiness Assessment (SARA) tool using the KoboCollect app. Data were cleaned and coded on Microsoft Excel 2016 and exported to Stata SE 12 for analysis. The level of significance was set at *p* < 0.05.

**Results:**

Overall, the MCH readiness score amongst PHC facilities was 47% (0.47 ± 0.18). About half (52%) of the facilities had necessary and relevant equipment. Health facilities located in urban areas had more medicines and commodities compared with those of rural areas (0.51 ± 0.16 vs 0.45 ± 0.17, *p* < 0.05). Primary health care facilities in Ekiti North I had an overall higher service readiness score (0.63 ± 0.19) compared with other federal constituencies (*p* < 0.001).

**Conclusion:**

The overall MCH-specific service readiness in Ekiti State was relatively low. Strategies to address the identified gaps for a smooth journey towards the achievement of Universal Health Coverage (UHC) are recommended.

## Introduction

Progressive efforts are in place towards the reduction in maternal and child mortality in the world.^1^ Despite all activities in place to sustain this reduction, the world is experiencing 216 maternal deaths per 100 000 live births, whilst the under-5 mortality rate is 42.5 deaths per 1000 live births.^1^ Regrettably, most of these deaths occur in low- and middle-income countries (LMICs), and more than half of global maternal deaths occur in sub-Saharan Africa.^2,3^ The 2018 Demographic and Health Survey (DHS) for Nigeria revealed a neonatal mortality rate as high as 39 per 1000 live births and a maternal mortality ratio of 512 per 100 000 live births.^4^ Although these indices depict a downward trend when compared with reports from past DHS, Nigeria still has one of the worst maternal, neonatal and infant health indices in the world. For each of the maternal deaths in Nigeria, about 18 other women suffer various morbidities, some with long-term socio-economic, physical and psychological consequences.^5^ Succeeding the Millennium Development Goals (MDG) are the sustainable development goals (SDG), with new targets set to be achieved. Universal Health Coverage (UHC) is required to achieve these goals, necessitating the assessment of health facility readiness to achieve UHC.^6^

Access to adequate infrastructure, diagnostic equipment, drugs and well-trained medical personnel are essential for the delivery of health care services.^7^ Poor budgeting and wrong use of funds affect the health care service delivery system, resulting in poor content and quality of health care services in Nigeria.^7^

[*T*]he principle of UHC reemphasizes distributional equity and efficiency in healthcare service delivery, through the provision of technical and financial support to healthcare facilities at all levels. These include the provision of services which are directly associated with the realization of several health-related targets in the SDG.^7^

The principle of UHC as part of the 2015 SDG makes countries set a goal to ensure that quality health services are available to everyone, everywhere, without catastrophic expenditure by the year 2030, and this goal can only be achieved through effective primary health care (PHC).^8^ Access to primary care services in LMICs is based on the availability of PHC facilities.

Primary health care was defined at the 1978 Declaration of Alma-Ata as:

[*E*]ssential health care based on practical, scientifically sound and socially acceptable methods and technology made universally accessible to individuals and families in the community through their full participation and at a cost that the community and country can afford to maintain at every stage of their development in the spirit of self-reliance and self-determination.^7,9^

Primary health care is the first point of contact for health services provision and a point of entry for the entire health system. Research has shown that access to PHC services is linked to better health outcomes.^9^ Hence access to PHC in the LMICs also hinges on the availability and readiness of the primary health centres. Primary health centres in the context of LMICs address geographical access and provide comprehensive services to the population, often with both curative and preventive components.^10^

One of the main functions of a health system is to ensure access to quality health services. Different components of service access include availability, affordability and acceptability.^11^ Service availability denotes the physical existence of the delivery of services and incorporates health infrastructure, core health personnel and aspects of service utilisation. Service *affordability* refers to how the service provider’s charges correspond to the client’s ability and willingness to pay for services, whilst service a*cceptability* refers to the extent to which the client is comfortable with the more incommutable characteristics of the provider and vice versa. These characteristics include the age, gender, social class and ethnicity of the provider (and of the client), including the diagnosis and type of coverage of the client.^12^

Service readiness, which is a prerequisite for service quality, is defined as the availability of mechanisms required to provide services in five domains that denote general service readiness. This refers to the overall capacity of health facilities to offer general health services that include possession of basic amenities, basic equipment, standard precautions for infection prevention, diagnostic capacity and essential medicines. On the other hand, specific-service readiness denotes the ability of health facilities to provide a particular service, and the ability to provide such service is measured through consideration of some tracer items which include trained staff, guidelines, equipment, diagnostic capacity, medicines and commodities.^13^

Nigeria operates a three-tiered health care delivery system, with a large percentage of health care delivery vested at the PHC level. The government has continued its efforts to decentralise healthcare services to the PHC centres to ensure that health services are located closer to the people and are also more affordable. This is expected to lead to the improvement of a wide range of health indices that affect the quality of life of the citizens.^14^ Primary health care was adopted in the 1988 National Health Policy as the cornerstone of the Nigerian health system as part of efforts to improve equity in access and utilisation of basic health services. Since then, PHC in Nigeria has evolved through various stages of development.^15^ In Nigeria, as well as other LMICs, huge gaps in the capacity and delivery of basic clinical care have been documented, as well as poor quality care, including poor care by health care providers.^16,17^ All these contribute to poor utilisation of PHC facilities amongst the public. It has been documented in Nigeria that PHC centres are poorly utilised, leading to failure to meet the goals of UHC.^7^

To achieve UHC, it is essential to assess the facility for service availability and readiness. Different researchers used different tools to collect the required information. Hence, the World Health Organization (WHO) developed a uniform and complete facility assessment tool titled Service Availability and Readiness Assessment (SARA), available in different countries.^18^ Therefore, this study was conducted to assess maternal and child health (MCH) services’ specific readiness by type and location of the facility, as well as to compare the readiness between the urban and rural PHC facilities in Ekiti State, Nigeria, using the SARA tool.

## Methods

### Study setting

Ekiti State is one of the 36 states in Nigeria, created on 01 October 1996. It lies south of Kwara and Kogi States, east of Osun State and limited by Ondo State in the east. Ekiti State has 16 local government areas (LGA), with a population of 3 270 798 (in 2016), a 2.3% annual growth rate and an area of 5887.89 square km.^19^ The state is located in the southwestern part of Nigeria between longitudes 40°51′ and 50°451′ east of the Greenwich meridian and latitudes 70°151′ and 80°51′ north of the equator. Ekiti State has 16 LGAs and six federal constituencies: Ekiti Central I (Ado, Irepodun, Ifelodun), Ekiti Central II (Ijero, Efon, Ekiti West), Ekiti North I (Ikole, Oye), Ekiti North II (Ido-Osi, Ilejemeje, Moba), Ekiti South I (Ekiti South-West, Ikere, Ise-Orun) and Ekiti South II (Ekiti East, Emure, Gbonyin). Each LGA has a minimum of 10 political wards and a total of 177 political wards in the state.^20^ The capital is located at Ado-Ekiti. The state is mostly agrarian, with small and medium-scale enterprises in urban and semi-urban areas. Health care is delivered through the public and private sectors. The PHC Development Agency (PHCDA) is an umbrella body controlling all the PHC workers’ activities in Ekiti State. The state currently has a total population of 4261 workers, which comprise technical and support staff. There are different categories of health care workers with varying levels of authority and supervision in respective LGAs.^21^

### Study design, population, sample size determination and data collection

This was a descriptive cross-sectional study conducted amongst the administrative heads (officers in charge) of primary health centres and primary health clinics in Ekiti State, Nigeria. The Ward Minimum Health Care Package (WMHCP) was developed to address the current strategy to deliver PHC services. Based on the ward health system in Nigeria, the three recognised facility types are health post, primary health clinic and PHC centre; however, health posts are not included amongst those qualified for the basic healthcare provision fund.^22^

Respondents for this study were officers who had been in charge of each of the facilities for at least six months. Each facility was also assessed using an observer checklist. The observer checklist was a component of the SARA tool, an instrument used to observe the availability of equipment in PHC facilities, and it was physically observed by the trained research assistants. The MCH components of the WHO SARA^13^ were adapted to focus mainly on service-specific readiness and availability for MCH. The survey was statewide, and the tool was administered by 16 trained research assistants for data collection. The questionnaire was interviewer administered using KoBo Toolbox (an open-source Android app for survey data collection). Data collection took place between August and October 2020. The questionnaire was pretested in similar semi-urban and rural PHC facilities in Lagos State before the commencement of the study. There are 177 wards in Ekiti state, with 177 PHC facilities under the Basic Health Care Provision Fund (BHCPF), with one PHC facility per ward. The minimum sample size (172) was determined using Cochran’s formula with a 95% confidence level and a proportion of 62% being a general service readiness index score of facilities based on a study on SARA using the same tool in Bangladesh.^23^ However, total sampling of all PHC facilities was done for better representation.

### Data management

Completed questionnaires were cleaned and coded using Microsoft Excel 2016 and were exported to Stata SE 12 (StataCorp LLC, College Station, TX, United States) for analysis. A score of 1 was awarded for a relevant item required for service delivery observed and 0 for its absence. Percentage and frequency distribution were used to present the various MCH services available at PHC facilities. The domain (basic equipment, diagnostics, staff training and guideline, medicine and commodities) score for each facility was carried out using the formula:

n/tracer items×100, [Eqn 1]

where *n* is the total number of an item available in each facility and the denominator is the number of indicator tracer items for each of the domains. Each tracer item for an individual facility was scored: yes = 1, otherwise = 0. The overall score for MCH service readiness was derived from this formula: the average score of the domains/number of domains.^24^ The domain score for each of the four domains (basic equipment, diagnostics, staff training and guideline, medicine and commodities), and the overall score for MCH readiness was represented as mean and standard deviation or mean percent score. Independent *t*-tests between the four domains across the urban and rural areas were used to assess the relationship between MCH-specific service readiness amongst health facilities in the urban and rural areas, whilst one-way analysis of variance (ANOVA) and independent *t*-tests were used to assess the association between the overall MCH-specific service readiness score across the different facility types, federal constituencies and urban or rural areas. The state and local government area headquarters were referred to as urban areas. The level of significance was set at a *p*-value less than 0.05.

### Ethical considerations

Ethical approval for the study was obtained from the Health Research and Ethics Committee of Lagos State University Teaching Hospital (ref. no. LREC/06/10/1424). Written informed consent was obtained from each respondent with an assurance of confidentiality of the information and their right to withdraw from the study at any point in time. They were made to understand that involvement was voluntary and had nothing to do with their employer.

## Results

### Distribution of primary health care facilities in Ekiti state

The majority (81.9%) of the PHC facilities were located in rural areas. About 18.6% were located in Ekiti Central II constituency and 94.9% were primary health centres (Table 1).

**TABLE 1:** Distribution of healthcare facilities in Ekiti state.

Variable	Frequency (*n* = 177)	%
**Location**		
Rural	145	81.9
Urban	32	18.1
**Federal constituency**		
Ekiti North I	23	13.0
Ekiti North II	32	18.1
Ekiti South I	32	18.1
Ekiti South II	32	18.1
Ekiti Central I	25	14.1
Ekiti Central II	33	18.6
**Type of health care facilities**		
Primary health centres	168	94.9
Primary health clinics	9	5.1

### Frequency of service-specific availability in primary health care facilities in Ekiti state

About two-thirds of the facilities (65%) offered family planning services, more than half (56.5%) stocked contraceptives at the service site, the majority (84.8%) offered antenatal care services and 68.9% offered foetal delivery services. Most (71.8%) offered assisted vaginal delivery services and about 79.1% carried out immediate and exclusive breastfeeding. About 10.7% of the facilities had national guidelines for prevention of mother to child transmission (PMTCT) services, 0.6% offered caesarean section and 1.1% had blood transfusion services. About 75.7% carried out hygienic cord care, a majority (96.6%) offered immunisation services and about 30.5% had guidelines for infant and young child feeding counselling (Table 2).

**TABLE 2:** Frequency of service-specific availability in primary health care facilities in Ekiti state.

Maternal and child health-specific services	Frequency	%
Offers family planning services	115	65.0
Offers family planning counselling to HIV-positive patients	37	20.9
Combined oestrogen–progesterone oral contraceptive pills	107	60.5
Offers progestin-only contraceptive pills	95	53.7
Offers combined oestrogen–progesterone injectable contraceptive pills	98	55.4
Offers availability of male condom	123	69.5
Offers availability of female condom	93	52.5
Availability of cycle bead for standard days method	19	10.7
Offers male sterilisation	3	1.7
Offers female sterilisation	6	3.4
Availability of intrauterine contraceptive device (IUCD)	72	40.7
Offers emergency contraceptive pills	46	26.0
Stock contraceptive commodities at the service site	100	56.5
Offers antenatal care services	150	84.8
Offers intermittent preventive treatment in pregnancy	133	75.1
Monitors hypertensive disorder in pregnancy	120	67.8
Provides iron supplementation	150	84.8
Provides folic acid supplementation	149	84.2
Provides tetanus toxoid immunisation	160	90.4
Offers services for the prevention of mother-to-child transmission	46	26.0
Provides ARV prophylaxis to HIV-positive pregnant women	17	9.6
Provides ARV prophylaxis to newborns of HIV positive pregnant women	14	7.9
Possess national guidelines for PMTCT	19	10.7
Provides nutritional counselling for HIV-positive pregnant women	38	21.5
Offers monitoring and management of labour using partograph	74	41.8
Offers corticosteroids in preterm labour	13	7.3
Offers foetal delivery services	122	68.9
Offer caesarean section	1	0.6
Assisted vaginal delivery	127	71.8
Manual removal of placenta	122	68.9
Neonatal resuscitation with bag and mask	56	31.6
Blood transfusion services	2	1.1
Carry out immediate and exclusive breastfeeding	140	79.1
Performs hygienic cord care	134	75.7
Performs thermal protection after delivery	134	75.7
Offer injectable antibiotics for neonatal sepsis	50	28.3
Offer immunisation services	171	96.6
Provide birth doses (e.g. hepB0, BCG, OPV0) both in the facility and outreach	172	97.2
Provides infant (under 1 year) vaccines both in facility and outreach	176	99.4
Provides adolescent and adult vaccines (e.g. HPV, tetanus) both in facility and outreach	158	89.3
Treatment of malaria in children	171	96.6
HIV guidance and counselling, testing for infants	36	20.3
Offers child growth monitoring	162	91.5
Diagnosis of malnutrition	144	81.4
Possess guidelines for infant and young child feeding counselling	54	30.5
Provide ORS for children with diarrhoea	167	94.4
Provide zinc supplementation for children with diarrhoea	155	87.6

ARV, antiretroviral; IUCD, intrauterine contraceptive device; HPV, human papilloma virus; HIV, human immunodeficiency virus; PMTCT, Prevention of mother-to-child transmission; ORS, oral rehydration solution; HepB0, hepatitis B zero; BCG, Bacillus Calmette-Guérin; OPV0, oral polio virus zero.

### Availability of medicines in primary health care facilities in Ekiti state

Vitamin A supplementation was the most readily available in almost all facilities in the present study, followed by gentamicin injection, amoxicillin and oxytocin injection (Table 3).

**TABLE 3:** Availability of medicines in primary health care facilities in Ekiti state.

Drugs	Frequency	%
Availability of hydralazine injections	15	8.5
Availability of metronidazole injections	55	31.1
Availability of azithromycin capsules or tablets or oral liquid	14	7.9
Availability of cefixime capsules or tablets	22	12.4
Availability of oxytocin injections	113	63.8
Availability of dexamethasone injections	31	17.5
Availability of betamethasone injections	3	1.7
Availability of sodium chloride injectable solution	43	24.3
Availability of intravenous solution with an infusion set	102	57.6
Availability of skin disinfectants	78	44.1
Availability of magnesium sulphate injectables	47	26.6
Availability of calcium gluconate injections	14	7.9
Availability of methyldopa tablets	58	32.8
Availability of nifedipine tablets	65	36.7
Availability of benzathine benzylpenicillin powder for injection	38	21.5
Availability of ampicillin powder for injection	56	31.6
Availability of gentamicin injections	132	74.6
Availability of antibiotic eye ointments for newborns	28	15.8
Availability of amoxicillin for treatment	130	73.5
Availability of vitamin A supplementation	171	96.6

### Specific-service readiness scores at primary health care facilities in Ekiti state

Overall, the MCH service readiness score amongst the facilities was 47% (0.47 ± 0.18). About half (52%) of the facilities had necessary and relevant equipment (Table 4).

**TABLE 4:** Specific-service readiness scores at primary health care facilities in Ekiti state.

Maternal and child health-specific services	Mean availability of items and s.d.	Percentage
Availability of equipment	0.52 ± 0.19	52.0
Availability of diagnostic services	0.44 ± 0.31	44.0
Availability of staff and training	0.47 ± 0.19	47.0
Availability of commodities and medicines	0.46 ± 0.17	46.0
Overall maternal and child health service readiness score	0.47 ± 0.18	47.0

s.d., standard deviation.

### Comparison of domain scores for maternal and child health-specific services readiness amongst primary health care facilities in urban and rural areas of Ekiti state

Primary health facilities located in the urban areas had more medicines and commodities compared with the rural areas (0.51 ± 0.16 vs 0.45 ± 0.17), with a statistically significant difference (*p* < 0.05) (Table 5).

**TABLE 5:** Comparison of domain scores for maternal and child health-specific service readiness amongst primary health care facilities in urban and rural areas of Ekiti state.

Service domain	Rural	Urban	*t*-test	*p*
Availability of basic equipment	0.51 ± 0.20	0.56 ± 0.16	1.32	0.094
Availability of diagnostic services	0.43 ± 0.31	0.44 ± 0.33	0.05	0.480
Availability of guidelines and staff training	0.46 ± 0.19	0.51 ± 0.18	1.27	0.102
Availability of commodities and medicine	0.45 ± 0.17	0.51 ± 0.16	1.81	0.036*

Note: Asterisk denotes statistical significance.

### Comparison of maternal and child health service readiness scores across types and locations of primary health care facilities

The health facilities in Ekiti North I had an overall higher service readiness mean score (0.63 ± 0.19) compared with others, with a statistically significant difference (*p* < 0.05) (Table 6).

**TABLE 6:** Comparison of maternal and child health service readiness scores across facility types and location.

Variable(*n* = 177)	Mean ± s.d.	Test statistics (*F*-ratio; *t*-test)	*p*
**Type of facility**			
Primary health centre	0.47 ± 0.19	0.28†	0.414
Primary health clinic	0.46 ± 0.11	-	-
**Location**			
Rural	0.46 ± 0.19	1.12†	0.132
Urban	0.50 ± 0.17	-	-
**Federal constituency**			
Ekiti North I	0.63 ± 0.19	7.75	0.000‡
Ekiti North II	0.50 ± 0.15	-	-
Ekiti South I	0.40 ± 0.21	-	-
Ekiti South II	0.50 ± 0.12	-	-
Ekiti Central I	0.38 ± 0.17	-	-
Ekiti Central II	0.44 ± 0.16	-	-

s.d., standard deviation.

† , *t*-test;

‡ , one way analysis of variance.

## Discussion

The study assessed MCH service’s specific readiness by type and location of the health facility with the comparison between urban and rural PHC centres in Ekiti State, Nigeria. The study found that most of the PHC facilities were in the rural areas of the state, with an almost equitable distribution amongst the federal constituencies. This distribution of the PHC facilities may be a step in the right direction towards the prevention of MCH deaths in rural areas. Studies in Nigeria have reported a higher incidence of maternal mortality in rural parts of the country as compared with urban areas.^25,26^

Some of the reasons that have been documented for the higher rate of maternal mortality in rural areas of Nigeria include the scantiness of healthcare infrastructure and health personnel for emergencies, especially during and post delivery.^17,27,28,29^ It is hoped that this will not be the case in Ekiti State, Nigeria, as more of the PHC facilities were located in rural areas. However, most of the hospitals to which emergencies can be referred by the rural health facilities are usually located in urban areas, and there may be limited opportunities for accessibility when needed because of various factors like the poor road network.^17,30^

One of the principles of UHC and health for all is to reach the most vulnerable persons and communities globally, with vital information and services to improve MCH and reduce the mortality rate.^11,31^ The majority of the health care facilities in this study were primary health centres, with only a few primary health clinics. This is because most of the primary health facilities are upgraded with more comprehensive service provisions.

Immunisation was the most frequently available service provided at the PHC facilities, and the majority of facilities had necessary vaccines in this study. This finding corroborates that of a similar study in Enugu State, Nigeria, which reported that newborn care and immunisation were the most frequently available services across facilities.^11^ This is in line with the expectation that PHC takes immunisation very seriously as a preventive health care service and is the closest form of health service to the masses. However, the finding differs from a study in Lagos, which reported treatment of ailments as the most commonly available service.^32^

Less than three-quarters of the facilities in this study provided family planning services. This finding is similar to a study in Bangladesh, which reported low family planning services.^6^ Intrauterine contraceptive devices (IUCDs) were provided by 40.7% of the PHC facilities in this study, which is higher than that reported by the study in Enugu, Nigeria, where only 18.3% of facilities provided IUCDs.^11^

Only a few of the PHC facilities in this study offered HIV guidance and counselling testing for infants and PMTCT. Only 10.7% had national guidelines for PMTCT, and 21.5% provided nutritional counselling for HIV positive pregnant women. The study in Enugu, Nigeria, found that HIV testing services were available in 68.0% of PHC centres, but only a quarter offered HIV treatment.^11^ Vitamin A supplementation was the most readily available medicine in almost all PHC facilities in the present study, followed by gentamicin injection, amoxicillin and oxytocin injection. The overall domain score for commodities and medicine in this study was 46.0%. The study in Enugu, Nigeria, also documented low rates of essential medicines in both urban and rural PHC facilities.^33^ This finding is also consistent with a study conducted in Tanzania, where 41.0% of facilities had essential medicines.^34^ The shortage of medicines might be because of an inadequate supply of medicines or irrational use of medicines by patients.

The overall MCH service readiness score for the four domains in this study was low at 47.0%. This was true for most domains: availability of diagnostic services (44.0%), availability of commodities and medicines (46.0%), availability of staff and training (47.0%) and availability of basic equipment (52.0%). This finding revealed wide gaps in the PHC facilities’ service delivery, which needs to be improved upon in Ekiti State, to enable the attainment of UHC for MCH care as globally envisaged. Bridging the gaps will help to improve the quality of MCH service delivery, leading to a reduction in mortality rate.

Comparing the domain scores for MCH-specific service readiness amongst PHCs in urban and rural areas in this study, the PHC facilities in the urban areas of the state had more medicines and commodities. Similarly, the specific-service readiness scores were higher in urban than in rural PHC facilities. The study in Enugu, Nigeria, reported a similar rural-urban disparity between PHC centres, with PHCs in rural areas five times less likely to have at least half of the recommended infrastructure or basic amenities and equipment compared with urban PHC centres. The study also documented low availability of medicine and supplies in rural PHC centres for maternal health services.^11^ Furthermore, a survey on service readiness, health facility management practices and delivery care utilisation in five states of Nigeria reported lower odds of health facility delivery in rural than in urban areas for the LGA mean index of management practices.^2^ In contrast, a study conducted in some middle and low-income countries reported that service availability and readiness were higher in rural facilities than in urban facilities.^35^ With this finding, Nigeria’s government ought to focus more on equity in the implementation of PHC services to reach the vulnerable citizens in the country, especially those in rural areas.

The study shows comparability of readiness by type of facility, with no statistically significant difference between the primary health centre and primary health clinic. However, we found a statistically significant difference in the overall mean of MCH-specific service readiness scores by federal constituencies. These regional differences between Ekiti North I and others may be explained by the fact that some primary health facilities in Ekiti North I enjoy collaboration with the Federal Medical Centre and State Hospital located in the same LGA for service availability and delivery.

This study uncovered critical gaps in service readiness and corroborated some findings of studies in Nigeria which showed major deficiencies in the PHC system. Previous studies had indicated deficiencies in various PHC facilities in Nigeria, from nonfunctional equipment for MCH services^2^ to almost half of PHC centres in rural areas failing to provide the MCH components.^36^ Similarly, a survey in an LGA in south-west Nigeria found that none of the facilities met the criteria for basic emergency obstetric care (BEmOC), with almost half of the facilities manned by unskilled health attendants; none of the health workers had ever been trained in lifesaving skills, and there was a widespread lack of BEmOC equipment and supplies.^37^ Another study found that most PHCs were unable to provide all BEmOC services and commonly lacked the required clinical staff.^38^ These findings point to the need for adequate provision of basic equipment, diagnostic services, guidelines, staff training, commodities and medicines in the PHCs. Whilst health facilities in rural areas need improved availability of commodities and medicine, urban areas should not be left out.

### Limitation

The cross-sectional nature of the study made it difficult to establish causality. However, this study is the first one known to us that has used the SARA tool (designed as a systematic approach for yearly validation of service delivery at the facility level) in the assessment of PHC facilities for MCH readiness in Ekiti State, Nigeria.

## Conclusion and recommendation

The overall MCH-specific service readiness in Ekiti State was relatively low. The low score was demonstrated in the domains of availability of diagnostic services and commodities and medicines in PHC facilities, with a statistically significant difference in medicines and commodities between rural and urban facilities. This gap demonstrated in the MCH-specific service readiness in Ekiti State requires the attention of policymakers and other stakeholders to devise strategies for successful and sustainable implementation of MCH services towards the achievement of UHC and SDG.
